# Fetal Growth Restriction in the Survivor Twin Following Spontaneous Demise in Monochorionic Pregnancy: A Case Report Highlighting Aplasia Cutis

**DOI:** 10.1002/ccr3.71227

**Published:** 2025-11-11

**Authors:** Ludovica Palandri, Daniela Casati, Donatella Fossa, Stefano Faiola, Arianna Laoreti, Mariano Lanna

**Affiliations:** ^1^ Fetal Therapy Unit “U Nicolini”—Buzzi Children's Hospital University of Milan Milan Italy; ^2^ U.O. Ostetricia e Ginecologia ASST Lariana— Ospedale Sant'Anna Como Italy

**Keywords:** abdomen/abnormalities, aplasia cutis congenita, fetal growth retardation, monochorionic fetus, twin pregnancy

## Abstract

Aplasia cutis congenita is a rare congenital malformation that may also occur as a complication of single fetal demise in a monochorionic twin pregnancy. Reporting such cases may improve prenatal detection rates in similar cases worldwide.

## 1 Introduction

Aplasia cutis congenita (ACC) is a rare congenital skin defect occurring in 1–3 per 10,000 live births. It involves the focal absence of the epidermis or dermis, affecting the scalp, the limbs, the trunk, or the extremities [1]. Cases of ACC have been reported as a result of ischemia in single survivors following fetal demise of a monochorionic (MC) twin [2]. However, descriptions of diagnostic signs are limited. This report discusses a misinterpretation of prenatal ultrasound signs of ACC in a surviving MC twin, initially mistaken for severe growth restriction.

## 2 Case History

A 25‐year‐old primigravida with a MC diamniotic twin pregnancy was referred to our unit at 19 weeks' gestation following the spontaneous demise of one twin at 14 weeks. At the time of diagnosis at 14 weeks, fetal anemia in the surviving twin could not be assessed, as the peak systolic velocity of the middle cerebral artery, a predictor of fetal anemia, is measurable only from 18 weeks' gestation [3]. Furthermore, the five‐week interval between the diagnosis at 14 weeks and referral at 19 weeks exceeded the timeframe for detecting acute hypovolemia [4]. At 19 weeks, a detailed ultrasound, including neurosonography, was performed, revealing no evidence of brain lesions. A fetal magnetic resonance imaging scan was subsequently planned for 21 weeks to confirm these findings, following both our clinical experience and existing literature [5, 6]. Subsequent follow‐up indicated fetal biometry with an estimated fetal weight below the 5th percentile, accompanied by normal Doppler velocimetry findings in the umbilical artery (UA). Biweekly ultrasounds were performed at the referral hospital until the third trimester. Due to severe static growth observed over two subsequent evaluations—despite normal UA Doppler findings—a cesarean section was deemed the safest option at 32 weeks' gestation, following corticosteroid administration to prevent neonatal respiratory distress syndrome. Unexpectedly, the neonate had an adequate weight for gestational age (1720 g), with no signs of hypoxia (APGAR score 8/9, arterial pH 7.21). The neonate presented with a large, butterfly‐shaped abdominal wall defect characterized by well‐defined margins, sparing the umbilicus, and extending along the posterior axillary line. The defect measured approximately 9 cm in height and 6 cm in width, encompassing a total area of 72 cm^2^. The upper abdominal musculature was absent, with some muscular presence localized at the peri‐umbilical region. The soft tissues were replaced by a translucent membrane, providing visualization of the underlying abdominal viscera and lower costal margins. The clinical presentation at birth was consistent with a diagnosis of aplasia cutis.

## 3 Methods

At 32 weeks of gestation, suspected fetal growth restriction—characterized by normal cephalic circumference and femoral length but an abdominal circumference below the 5th percentile—prompted cesarean delivery. The diagnosis of ACC was made exclusively at birth, based on the clinical presentation. The neonate underwent triweekly dressings as part of a protocol established by the plastic surgeons at the reference hospital to ensure optimal outcomes. The dressings included non‐adherent, absorbent gauze, amorphous hydrogel, and an ointment containing collagen and hyaluronic acid. Follow‐up was regular, with excellent outcomes.

**FIGURE 1:**
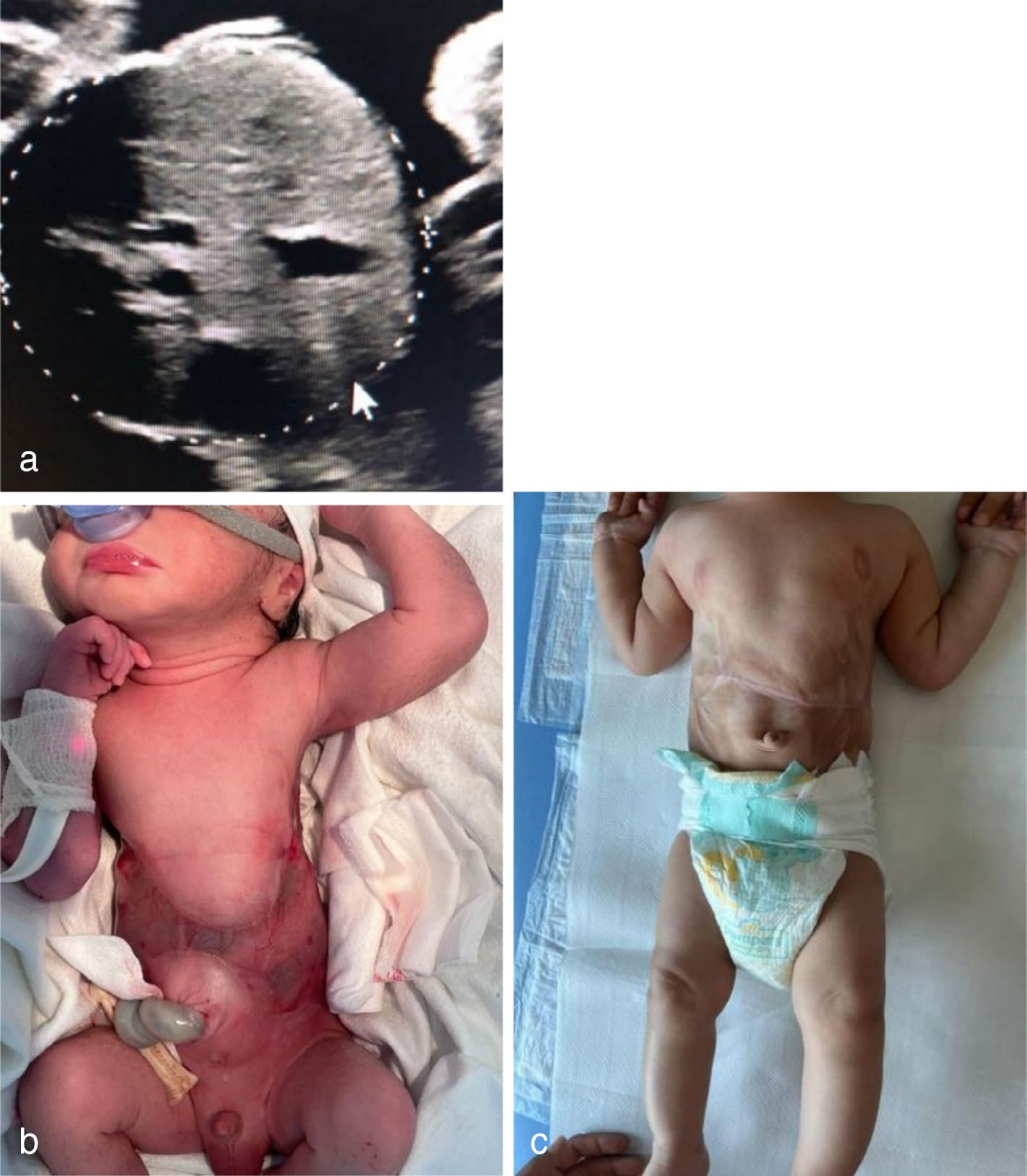
Aplasia Cutis Congenita due to the intrauterine death of a twin complicating a monochorionic diamniotic twin pregnancy (a) abdominal circumference at 30 weeks' gestation, where is not evident the outer layer; (b) the newborn shows a symmetrical abdominal wall lesion with a H‐shaped distribution, with a translucent membrane covering the abdominal organs; (c) reconstruction of the abdomen after medical treatment.

Only the retrospective evaluation of fetal biometry ultrasound images revealed the absence of external skin layers at the abdominal circumference measurement site (Figure 1a), corresponding to the H‐shaped skin defect observed postnatally (Figure 1b). A successful medical correction was performed (Figure 1c).

## 4 Conclusion and Results

The diagnosis of ACC was rapidly confirmed at birth, and the neonate was successfully treated with abdominal reconstruction. Follow‐up revealed normal neurodevelopmental milestones: independent sitting at 10 months, assisted standing at 12 months, and autonomous walking at 18 months.

## 5 Discussion

This is the first documented case of ACC following a single fetal demise in an MC twin pregnancy at our Fetal Therapy Unit over the past 20 years. ACC presents with a wide range of manifestations. The Frieden classification, established in 1986, categorizes ACC based on localization and associated abnormalities, including its association with fetus papyraceus or placental infarcts [7]. Subsequent cases of ACC have been reported after a single twin demise, most with abdominal defects managed through topical treatments or plastic surgery in severe cases [8]. While the increased risk of brain injury in surviving MC twins is well‐documented, with lesion rates between 18% and 35% [5, 6], other consequences of acute hypovolemia, such as ACC, are less reported. ACC occurrence is linked to early gestational ages at the time of single demise and selective termination in MC twins [9]. Standard prenatal evaluation of surviving twins often focuses on brain injury, with minimal attention to ACC. In one case, the absence of a hyperechogenic line between the abdomen and amniotic fluid in a surviving MC twin was considered a possible marker for truncal ACC, later confirmed postnatally. The authors recommend including this condition in differential diagnoses when abdominal organs appear exposed in surviving MC twins, despite the difficulty of recognizing such signs [10]. Another report investigating growth restriction in a surviving MC twin found elevated amniotic fluid concentrations of alpha‐fetoprotein and acetylcholinesterase, suggestive of prenatal ACC diagnosis [11]. While amniotic fluid protein assays could not be considered standard care for MC twin survivors due to the rarity of ACC and risks associated with invasive procedures, they may support prenatal suspicion in unexplained growth restriction. Reporting such cases could improve ACC detection rates, particularly in differential diagnoses for growth restriction in MC twins. Prenatal diagnosis may not improve outcomes, but could inform parents about medical steps for the newborn and facilitate optimal delivery planning. The main limitation of this report is the lack of prenatal suspicion, leading to unnecessary preterm delivery without adverse consequences for the newborn. This oversight likely stemmed from prioritizing fetal brain injury risks over other potential complications in surviving twins. Additionally, placental necrosis in single‐survivor twins can contribute to growth restriction. However, in the absence of abnormal UA Doppler findings, detailed observation of the fetal trunk and abdomen could help identify skin layer absence and allow pregnancy prolongation without increased fetal death risk.

## Author Contributions

**Ludovica Palandri:** conceptualization, data curation, formal analysis, software, writing – original draft. **Daniela Casati:** validation, visualization. **Donatella Fossa:** investigation, resources. **Stefano Faiola:** validation, visualization. **Arianna Laoreti:** validation, visualization. **Mariano Lanna:** funding acquisition, methodology, project administration, supervision, writing – review and editing.

## Ethics Statement

The ethical committee approved the study (n 612/2023).

## Consent

Written informed consent was obtained from the patient to publish this report in accordance with the journal's patient consent policy.

## Conflicts of Interest

The authors declare no conflicts of interest.

## Data Availability

The data that support the findings of this study are available from the corresponding author upon reasonable request.
